# An investigation of pain intensity patterns and psychosomatic symptoms among the cardiac patients admitted to an Iranian hospital

**DOI:** 10.22088/cjim.12.2.167

**Published:** 2021-03

**Authors:** Mostafa Bahremand, Afsaneh Shahbazirad, Gholamreza Moradi, Abdolhamid Zokaei

**Affiliations:** 1 1.Cardiovascular Research Center, Kermanshah University of Medical Sciences, Kermanshah, Iran

**Keywords:** Heart diseases, Psychosomatic medicine, Pain

## Abstract

**Background:**

Heart diseases are the main reasons of death in the developed countries. This study was conducted with the aim of investigating the patterns of pain intensity and psychosomatic symptoms among cardiac patients.

**Methods:**

This is a descriptive study and the statistical population included all the cardiac patients who were admitted to an Iranian Hospital (Kermanshah-Imam Ali Hospital) during 2018 (From July to November) 250 people were selected out of the population using convenience sampling and 180 patients answered pain intensity assessment tools, the patient health questionnaire (PHQ-15) and the patient demographic information form.

**Results:**

The results of this study indicated moderate (52.2%), low (36.1%) and high (11.7%) levels of pain intensity plus mild (4.4%), moderate (28.3%) and severe (67.2%) psychosomatic symptoms with the most common psychosomatic symptoms being chest pain (52.8%), fatigue (52.8%), shortness of breath (49.4%), heart palpitation (47.8%).The results of chi-square test showed a no significant relationship between psychosomatic symptoms and variables of age, marital status, drug abuse and pain intensity (*p*> 0.05), however, significant relationship was found between psychosomatic symptoms and gender (*p*< 0.001). Moreover, there was no significant relationship between pain intensity and age, gender, marital status and drug abuse at the level of (*p*>0.05).

**Conclusion:**

Psychosomatic symptoms and pain intensity are associated with certain functional disorders and health care, therefore, identifying psychosomatic symptoms and pain intensity is very useful in assessing the effectiveness of clinical approaches on cardiac patients.

Heart diseases are the main reasons of death in the developed countries. Although death rate from heart diseases has reduced in recent decades, it still accounts for about one third of all the death over the age of 35, which causes 17 million people death each year; it is predicted if no prevented actions are taken till 2020, the death rate will reach 24.8 million (1).Somatization is in fact the relationship between medically unexplained physical symptoms and mental disorders and health behaviors and it exists among at least 10 to 15 percent of primary care patients (2).These patients have recurring physical symptoms which cause an increase in using medical care, functional disorders and a decrease of life quality (3).Although cardiac patients are more likely to show cardiac symptoms from among psychosomatic symptoms (e.g. angina pectoris), yet few studies have been conducted about the prevalence of psychosomatic symptoms and its effects on their health (4).

Researches of Lambert &Dawood (5) showed the association of psychosomatic with cardiac failures. Kohlmann et al. suggest that the severity of psychosomatic symptoms can be considered as a risk factor for the suffering and death among cardiac patients (6). A study by Nabi et al. also indicated that psychosomatic symptoms would increase the risk of heart disease in women (7). In prior studies, Kohelmann et al. found out that psychosomatic symptoms of cardiac patients mostly include sleep disorders (76.7%), fatigue (70.8%), pain in the arms and knees (63%), back pain (61.2%), shortness of breath (59.4%) and more than 50 percent of the patients reported more psychosomatic symptoms (6), while according to Lovlien’s study, chest pain (45%) and fatigue (62%) have been the most reported symptoms among cardiac patients (8) and a study by McSweeney indicated that symptoms of fatigue (73%), sleep disorders (50%) and chest pain (37.7%) are the most common symptoms among cardiac patients (9).

Another important variable is pain intensity which is a sensory, emotional, cognitive and social experience and concerns damage to textures (10). There is a strong association between chronic pain and general health and studies show a direct relationship between pain intensity and poor health regarding social, psychological and physical aspects (11). Pain as an independent factor, is directly associated with poor health, heart disease (12, 13, 14), severe disabilities and lower wellbeing (15). Pain is highly prevalent among cardiac patients (16) and it has been reported in 40 to 70 percent of hospitalized cardiac patients (17). Goodlin et al. have also found a relationship between heart disease and pain (18).

Identifying psychosomatic symptoms and the intensity of pain play a significant role in measuring the effectiveness of therapeutic approaches on patients, thus, assessing this issue requires further investigation and examination. Therefore, considering the significance of the issue and the cultural and geographical differences that must be given priority in medical and psychological researches, the current study has tried to investigate pain intensity patterns and psychosomatic symptoms among cardiac patients of Iranian hospital during 2018.

## Methods

**Study design and setting: **This descriptive cross-sectional study which its statistical population consists of referring patients to an Iranian Hospital (Kermanshah-Imam Ali hospital) in 2018 (From July to November). Sampling was done through the available sampling -250 people were selected among the hospitalized patients in the hospital. The number of 180 patients answered the questions. The criteria for entering the study included the ability to answer the questions,the willingness to cooperate in the study; and the patients undergoing angiography and waiting for angiography while the criteria for withdrawing from the study included psychiatric illnesses and patients undergoing surgery and unwillingness to answer the questions. Ethical principles of being a volunteer for research; observance of individual rights and freedom of participants for non-willingness to fill out questionnaires. And it was also assured that the participants’ information is reported in groups.

**Study instruments: **Following tools have been used for gathering data in this study.

-Visual analogue scale (VAS) for pain measurement is presented as a 10 cm line with the left end (0) indicating no pain and the right end (8) indicating the worst pain. The scores of 1 to 3, 4 to 7, and 8 to 10 suggest mild, moderate and severe pain, respectively (19). Reliability of this scale has been confirmed by Kroenke et al. (20).

-The Patient Health Questionnaire (PHQ-15): it is a self-related instrument which contains 15 items which are based on the prevalence of various somatic symptoms seen in patients presenting to the outpatient setting. Ratings are done by considering the last 4 weeks into account, and each item is rated as 0 (not bothered at all), 1 (bothered a little), or 2 (bothered a lot) with the total score ranging from 0 to 30. On the basis of the total score, the severity of somatic symptoms is classified as mild (0-4), moderate (5-9), and severe (>10) (20). The full version of PHQ has been translated into Hindi and has been shown to have good psychometric properties (21). Also, Lee cited, Cronbach's alpha 0.79 for this questionnaire (22).

**Data Analysis: **In addition to descriptive statistics, inferential statistics such as chi-square test was used to analyze the data. The software used to analyze the data was SPSS Version 20

## Results

**Patient characteristics: **The current study has been conducted in 3 months, from April to June 2018. Among all 250 hospitalized patients, 180 individuals completed the questionnaire. The average age of these patients was 59.98±12.51 and 48.9 percent of them were females and 51.1 percent were males. The majority of the participants were married (75%), 43.9% of them were housewives and 36.7% worked as freelancer. 87.8 % reported no drug abuse (cigarette, opium, etc.) while 10.6 % resigned and 1.7% abused drugs. Regarding the measurement of pain intensity, 52.2% reported moderate pain. 36.1% had mild pain and 11.7% suffered severe pain. The results also indicated that 4.4% of the cardiac patients reported mild psychosomatic symptoms, 28.3 moderate symptoms and 67.2% severe symptoms. The most frequent psychosomatic symptoms were chest pain (52.8%), fatigue (52.8%) shortness of breath (49.4%), heart palpitation (47.8%), arm and leg pains (42.8%), back pain (25%), sleep disorder (21.7%), headache (19.4%), stomach pain (18.9%), dizziness (15%), nausea (13.9%), constipation and diarrhea (12.8%), debility (9.4%), painful intercourse (6.7%) and stomach cramp in women (2.8%). As shown in table 1, the results of chi-square test (*P*>0.05) indicated that psychosomatic symptoms are not significantly associated with age (X^2^=1.917), marital status (X^2^=3.733) and drug abuse (X^2^= 1.350). But the results indicated a significant relationship between psychosomatic symptoms and gender (P<0.001 and X^2^= 18.736). The results of Cramer’s V test (0.323) also support the significant relationship between these two variables. The results of chi-square test (*P*>0.05) shown in table 2 indicated that pain intensity is not significantly associated with age (X^2^= 2.466), gender (X^2^= 4.108), marital status (X^2^= 0.035) and drug abuse (X^2^= 4.98).

**Table1:** The study of psychosomatic symptoms with respect to demographic variables

Cramer's V	P-value	X^2^	Somatic Symptom			Variable	
			Much (>10)	Medium (5-9)	Weak (0-4)		
**0.073 (NS)**	0.7	1.917	0 25 66	1 17 49	0 5 17	18-29 years 30-50 years >50	Age
*0.323	0.001	18.736	59 32	22 45	7 15	Female Men	Gender
**0.14 (NS)**	0.15	3.733	28 63	14 53	3 19	Single Married	Married status
**0.06 (NS)**	0.8	1.350	1 80 10	1 59 7	1 19 2	Yes No Leave	Addiction

*P<0.001

NS: Not Significant

**Table 2:** Investigating pain intensity according to demographic variables

			Pain			Variable	
Cramer's V	P-value	X^2^	Much (8-10)	Medium (4-7)	Weak (1-3)		
**0.083 (NS)**	0.6	2.466	0 4 17	0 25 69	1 18 46	18-29 years 30-50 years >50	Age
**0.151(NS)**	0.1	4.108	14 7	47 47	27 38	Female Men	Gender
**0.014 (NS)**	0.9	0.035	5 16	24 70	16 49	Single Married	Married status
**0.118 (NS)**	0.28	4.98	0 17 4	2 80 12	1 61 3	Yes No Leave	Addiction status

NS: Not Significant

## Discussion

The aim of this study was to investigate patterns of pain intensity and psychosomatic symptoms among cardiac patients and according to the results 67.2 % of the patients reported severe psychosomatic symptoms and chest pain, fatigue, shortness of breath, rapid heartbeat, arm and leg pain and back pain were respectively the most common symptoms observed among the patients. Our results are supported by researches of Lambert &Dawood (5) and by Kohlmann et al.’s study (6) which has concluded that the most common psychosomatic symptoms are sleep disorder, fatigue, pain in arm and knee, backache and shortness of breath. Our findings are also in agreement with Nabi et al. (7) studies that found a relationship between cardiac patients and psychosomatic symptoms. Moreover, studies conducted by Lovlien (8) and MCSweeney (9) support our findings to some extent.

Because of the nature of their illness, and their constant concerns and worries about the occurrence of symptoms such as rapid heartbeat, short breath etc. cardiac patients seem to be more vulnerable to psychosomatic conditions. Furthermore, because of their constant experience of stress and vulnerability of a particular part of their body, these patients are more likely to experience psychosomatic symptoms. These patients’ understanding and perception about their environment are influenced by their physical condition and this eventually makes them more likely to report psychosomatic symptoms. The constant stress and anxiety experienced by the patients would lead them to passivity and despair thus makes them more likely to physicalize their symptoms.

We also found out that psychosomatic symptoms are not significantly associated with age, marital status, drug abuse and pain intensity. Our findings are in agreement with those of Soltani et al. (23) who asserted that there is no relationship between personal characteristics and symptoms such as nausea, loss of appetite, shortness of breath, gastronomic problems etc. Our findings are also to a certain extent consistent with the findings of Maharaj et al. (24). Since cardiac patients experience warning physical signs of the disease regardless of their age and marital status, they feel more vulnerable and less self-controlled; therefore, the emergence of psychosomatic symptoms among these patients cannot be influenced by demographic factors. The results also suggested a relationship between psychosomatic symptoms and gender which are in agreement with the findings of Maharaj et al.’s study (24). Compared to men, women seem to be more likely to develop psychosomatic symptoms. Similarly, studies have shown that psychosomatic symptoms are considerably higher among women than men (5 to 1) (25). The reason might be that due to their gender characteristics and accepted social and cultural stigmas, women are more likely to show such symptoms, though men might manifest these symptoms in other ways. 

Majority of the patients had reported moderate pain intensity which is consistent with the studies by Walke et al (17), Goodlin et al. (18). The results of meta-analysis by Fayaz et al. (26) also indicated a relationship between pain and heart disease. Moreover, constant dealing of patients with cardiac physical symptoms would lead to a better comparison and self-assessment of the experienced pain. The biological response to acute pain includes activation of the systematic nervous system which may contribute to a reduction in pain sensitivity that has been demonstrated both clinically and experimentally (27). On the other hand, the frequent exposure of patients with physical symptoms leads to a better self-assessment of the pain experienced.

This study found no significant relationship between pain intensity and gender, age, marital status and drug abuse. This finding is to some extent in agreement with a study by Helfer (28). It seems that the people’s experience of pain intensity as a warning sign of heart disease is not related to their gender and age background and other factors such as personality type, anxiety levels, personal experiences and expectations might play a role requiring further examination.

One of the limitations of this study was gathering information using questionnaires and participants’ self-report which might be affected by factors such as their tendency to give socially accepted answers. Another limitation concerns the participants’ condition, that is the questionnaires might have been given to them at a time when they were tired or in low mood and could not pay enough attention and thus, disturbing factors might affect their answers and bias to the results of the study. Further studies with larger groups and the use of personal interviews are recommended to achieve better results. More studies are also recommended to identify the moderating role of personal factors in the emergence of somatic symptoms and pain intensity of cardiac patients.

Finally, the findings of the present study can provide a useful practical base for organizing educational and health programs for the patients. Similarly, diagnosing and identifying psychosomatic symptoms and pain intensity can be very useful in assessing the effectiveness of clinical approaches on cardiac patients.

**Ethical approval and consent to participate**

All participants obtained informed consent form and this study was conducted and approved by the Ethics Council of Kermanshah University of Medical Sciences (IR.KUMS.REC.1396.580)
